# Supplementary material for: Functional locus coeruleus imaging to investigate an ageing noradrenergic system
Source: Commun Biol. 2024 Jun 27;7:777. doi: 10.1038/s42003-024-06446-5 (PMC11211439; doi:10.1038/s42003-024-06446-5)
Supplement: Supplementary file 2 — Supplementary Information [file 42003_2024_6446_MOESM2_ESM.pdf]

Supplementary Information

Functional locus coeruleus imaging to investigate an ageing noradrenergic system

Mareike Ludwig[1,2,3], Yeo-Jin Yi[1,3], Falk Lüsebrink[3,4,5], Martina F. Callaghan[6], Matthew J. Betts[1,2,3], Renat Yakupov[3], Nikolaus Weiskopf[6,7,8], Raymond J. Dolan[6,9], Emrah Düzel[1,3,10]*, Dorothea Hämmerer[1,2,3,6,10,11]*

[1]Institute of Cognitive Neurology and Dementia Research, Otto-von-Guericke University Magdeburg, Magdeburg, Germany;[2]CBBS Center for Behavioral Brain Sciences, Magdeburg, Germany; [3]German Center for Neurodegenerative Diseases (DZNE), Magdeburg, Germany; [4]Biomedical Magnetic Resonance, Faculty of Natural Sciences, Otto-von-Guericke University, Magdeburg, Germany; [5]NMR Methods Development Group, Max Planck Institute for Human Cognitive and Brain Sciences, Leipzig, Germany; [6]Wellcome Centre for Human Neuroimaging, UCL Queen Square, Institute of Neurology, University College London, 12 Queen Square, London WC1N 3AR, UK; [7]Department of Neurophysics, Max Planck Institute for Human Cognitive and Brain Sciences, Stephanstraße 1a, 04103 Leipzig, Germany; [8]Felix Bloch Institute for Solid State Physics, Faculty of Physics and Earth Sciences, Leipzig University, Linnéstraße 5, 04103 Leipzig, Germany; [9]Max Planck University College London Centre for Computational Psychiatry and Ageing Research, London WC1B 5EH, United Kingdom; [10]Institute of Cognitive Neuroscience, University College London, London WC1N 3AZ, United Kingdom; [11]Department of Psychology, University of Innsbruck, Innsbruck, Austria; *shared last authorship

To whom correspondence should be addressed: Mareike Ludwig, Institute of Cognitive Neurology and Dementia Research, Otto-von-Guericke University Magdeburg, Magdeburg, Germany. Email: mareike.ludwig@med.ovgu

Anatomical masks

a **b**

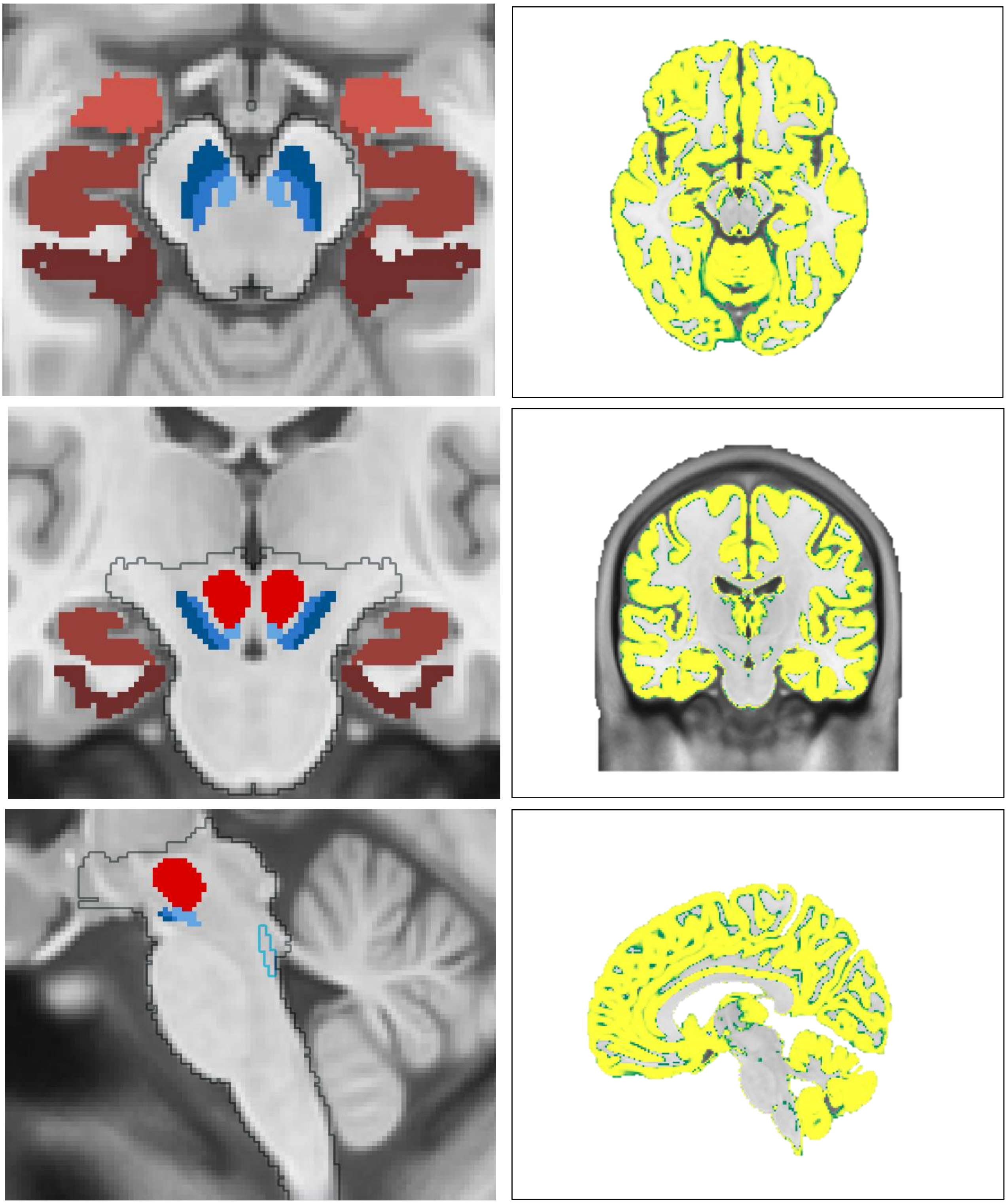

Supplementary Figure 1. Anatomical masks for second-level analyses for nuclei in the brainstem and midbrain (**a**): *substantia nigra pars reticulata (SNr,* dark blue*), substantia nigra pars compacta (SNc,* middle blue*), ventral tegmental area (VTA,* brighter blue*), red nucleus* (red), amygdalae (rose), hippocampi (middle rose), para-hippocampi (dark rose) and locus coeruleus (turquoise outline) are shown within brainstem mask (grey outline). Additionally (**b**) grey matter mask (yellow-green) and **(b)** brainstem mask (grey outline) were used as an implicit mask in second-level analyses. Masks are presented in axial, coronal and sagittal view (row 1-3).

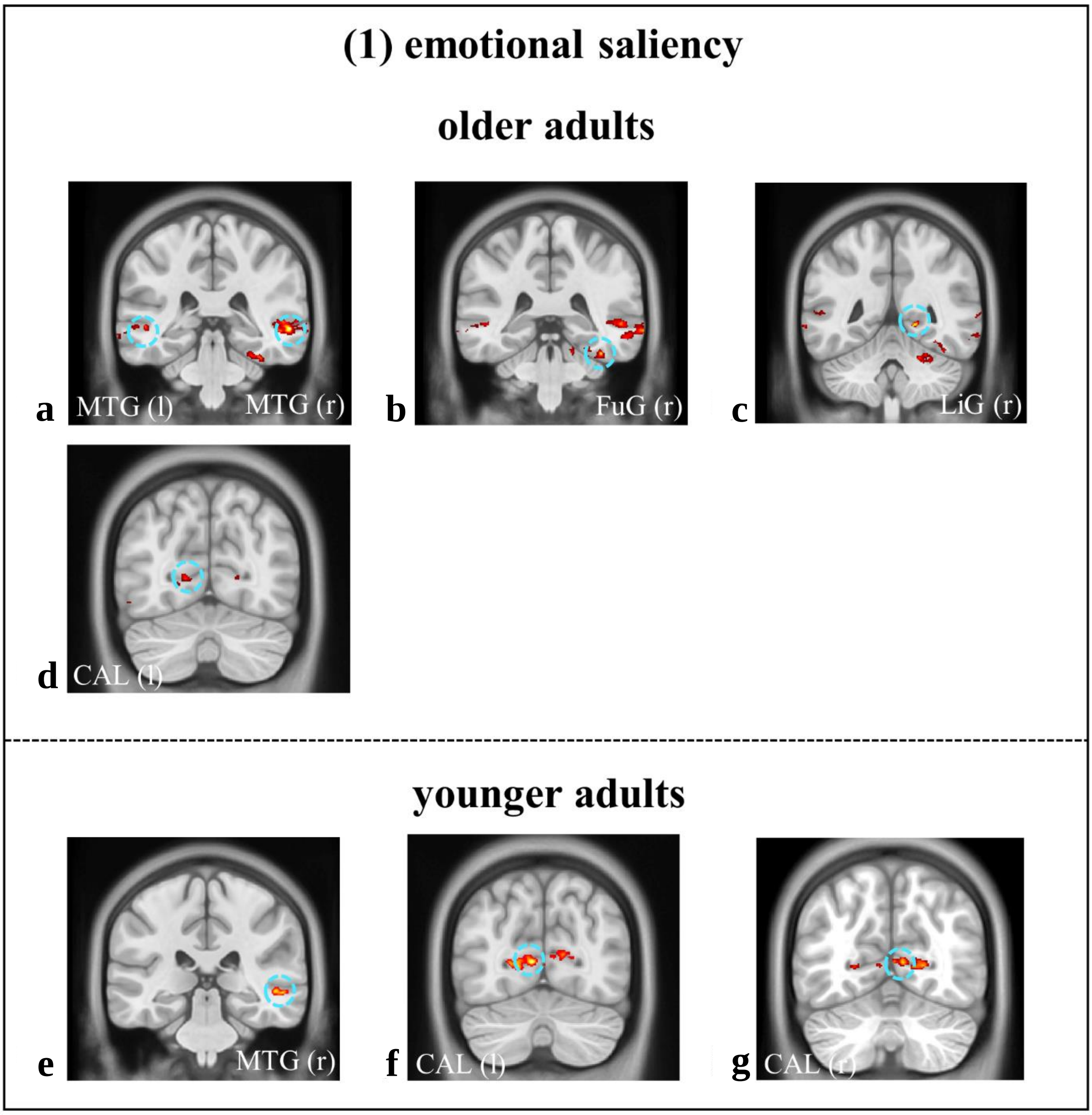

Supplementary Figure 2. Activations in cortical areas for **(1) emotional salience** (red-yellow) for (**a-d**) older adults and (**e-g**) younger adults. Turquoise circles highlight the corresponding significant activations, threshold of $p < 0.005$. Abbreviations: CAL calcarine cortex, FuG fusiform gyrus, l left, LiG lingual gyrus, MTG middle temporal gyrus, r right.

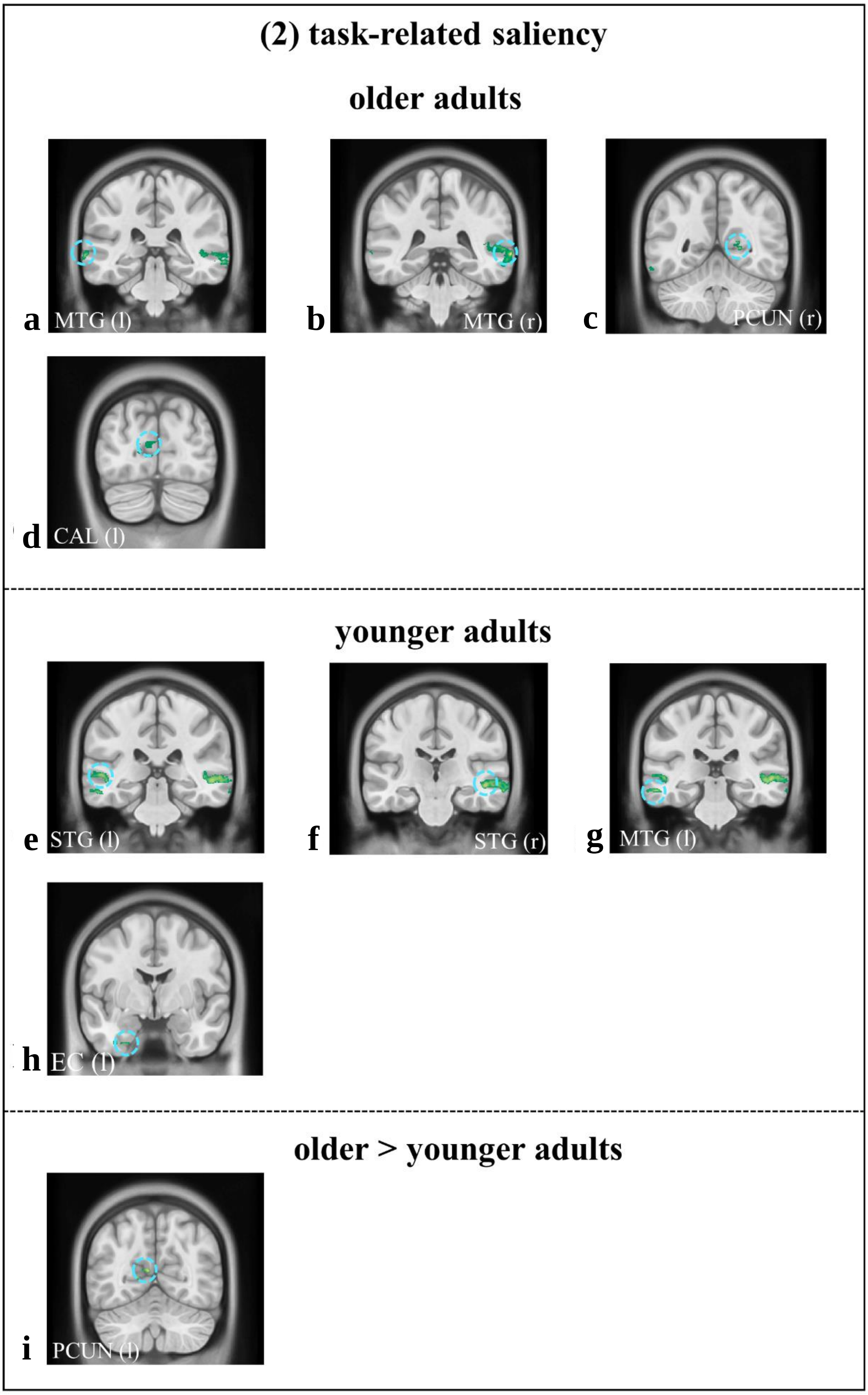

Supplementary Figure 3. Activations in cortical areas for **(2) task-related salience** (green-yellow) for (**a-d**) older adults (**e-h**) younger adults, (**i**) older > younger adults. Turquoise circles highlight the corresponding significant activations, threshold of $p < 0.005$. Abbreviations: CAL calcarine cortex, EC entorhinal cortex, l left, MTG middle temporal gyrus, PCUN precuneus, r right, STG superior temporal gyrus.

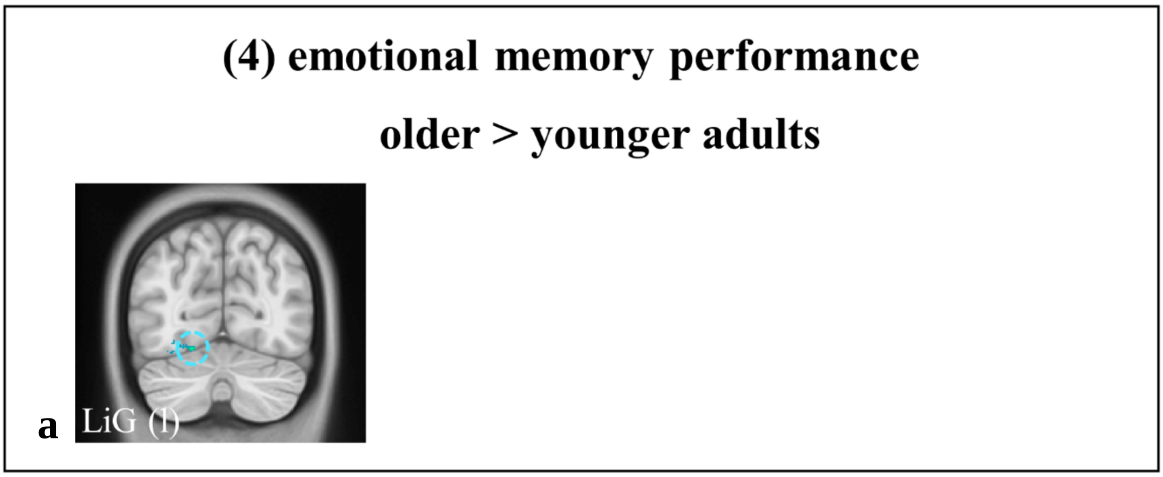

Supplementary Figure 4. Activation in cortical areas for **(4) emotional memory performance** (blue-green) for (**a**) older > younger adults. Turquoise circle highlights the corresponding significant activation, threshold of $p < 0.005$. Abbreviations: l left, LiG lingual gyrus.

The following **Supplementary Tables** report **(1-14)** **brainstem and midbrain activations** as well as **(15-21,23)** **cortical activations** for **(1)** emotional salience, **(2)** task-related salience, **(3)** memory performance and **(4)** emotional memory performance. Abbreviations: CAL *calcarine cortex*, CBM *cerebellum*, EC *entorhinal cortex*, FuG *fusiform gyrus*, HPC *hippocampus*, ITG *inferior temporal gyrus*, LC *locus coeruleus*, LiG *lingual gyrus*, MTG *middle temporal gyrus*, PCUN *precuneus*, red Ncl *red nucleus*, SNc *substantia nigra pars compacta*, SNr *substantia nigra pars reticulata*, VDC *ventral diencephalon*, VTA *ventral tegmental area*, WM *white matter*.

brainstem & midbrain activations

(1) emotional salience: loss feedback > gain feedback

older adults

						cluster					peak				
	region	x (mm)	y (mm)	z (mm)	Z (max.)	C	p (FWE corr.)	p (FDR corr.)	K (equiv.)	p (unc.)	T	p (FWE corr.)	p (FDR corr.)	Z (equiv.)	p (unc.)
p < 0.005	**LC (l)**	-5	-38	-26	4.11	2	0.04	0.55	15	0.29	4.11	0.01	0.23	3.48	< 0.001
	LC (r)	4	-37	-22	3.05	2	0.07	0.55	5	0.55	3.05	0.08	0.88	2.75	0.003
	SNr (l)	-6	-18	-18	4.46	3	0.31	0.66	20	0.22	4.46	0.09	0.17	3.69	0.001
	VDC (r)	4	-19	-6	3.02	3	0.75	0.82	1	0.82	3.02	0.71	0.99	2.72	0.003
	VDC (l)	-10	-23	-16	2.84	3	0.75	0.82	1	0.82	2.84	0.81	0.99	2.59	0.004
p < 0.003	**LC (l)**	-5	-38	-26	4.11	1	0.02	0.29	12	0.29	4.11	0.01	0.12	3.48	< 0.001
	SNr (l)	-6	-18	-18	4.46	1	0.24	0.23	15	0.23	4.46	0.09	0.08	3.70	< 0.001

Supplementary Table 1. Activations in older subjects for emotional salience with applying a **'brainstem mask'** and small volume image of **'LC meta mask'** or **'SNredVTA mask'** cluster threshold p < 0.005 and p < 0.003 with no adjusted FDRc.

older adults > younger adults

						cluster					peak				
	region	x (mm)	y (mm)	z (mm)	Z (max.)	C	p (FWE corr.)	p (FDR corr.)	K (equiv.)	p (unc.)	T	p (FWE corr.)	p (FDR corr.)	Z (equiv.)	p (unc.)
p < 0.005	**LC (l)**	-5	-37	-25	3.40	2	0.06	0.69	6	0.55	3.40	0.02	0.34	3.20	< 0.001
	**LC (r)**	4	-37	-22	3.14	2	0.08	0.69	3	0.69	3.14	0.04	0.34	2.98	0.001
	VDC (r)	4	-19	-6	2.74	1	0.72	0.83	1	0.83	2.74	0.74	0.90	2.63	0.004
p < 0.003	**LC (l)**	-5	-37	-25	3.40	2	0.04	0.72	4	0.59	3.40	0.53	0.53	3.20	< 0.001
	**LC (r)**	4	-37	-22	3.14	2	0.05	0.72	2	0.72	3.14	0.53	0.53	2.98	0.001

Supplementary Table 2. Activations in older > younger subjects for emotional salience with applying a **'brainstem mask'** and small volume image of **'LCmeta mask'** or **'SNredVTA mask'** cluster threshold p < 0.005 and p < 0.003 with no adjusted FDRc.

(1) emotional salience: loss feedback > gain feedback

younger adults

						cluster					peak				
	region	x (mm)	y (mm)	z (mm)	Z (max.)	C	p (FWE corr.)	p (FDR corr.)	K (equiv.)	p (unc.)	T	p (FWE corr.)	p (FDR corr.)	Z (equiv.)	p (unc.)
p < 0.005	SNc (r)	10	-21	-11	4.35	5	0.41	0.75	14	0.34	4.35	0.06	0.16	3.75	< 0.001
	SNr (r)	9	-16	-17	4.33	5	0.52	0.75	8	0.48	4.33	0.07	0.16	3.74	< 0.001
	SNr (l)	-10	-14	-15	3.73	5	0.28	0.75	24	0.22	3.73	0.22	0.38	3.32	< 0.001
	SNr (r)	9	-13	-11	3.08	5	0.65	0.75	3	0.68	3.08	0.59	0.67	2.83	0.002
	SNc (r)	5	-20	-19	3.00	5	0.68	0.75	2	0.75	3.00	0.64	0.67	2.76	0.003
p < 0.003	SNc (r)	10	-21	-11	4.35	5	0.29	0.81	12	0.32	4.35	0.06	0.23	3.75	< 0.001
	SNr (r)	9	-16	-17	4.32	5	0.43	0.81	5	0.53	4.33	0.07	0.23	3.74	< 0.001
	SNr (l)	-10	-14	-15	3.72	5	0.24	0.81	16	0.25	3.73	0.22	0.55	3.31	< 0.001
	SNr (r)	9	-13	-11	3.08	5	0.57	0.81	1	0.81	3.08	0.59	0.96	2.83	0.002
	SNc (r)	5	-20	-19	3.00	5	0.57	0.81	1	0.81	3.00	0.64	0.96	2.76	0.003

Supplementary Table 3. Activations in younger subjects for emotional salience with applying a **‘brainstem mask’** and small volume image of **‘LC meta mask’** or **‘SNredVTA mask’** cluster threshold p < 0.005 and p < 0.003 with no adjusted FDRc.

older adults

(2) task related salience: reversal > no reversal

	region	x (mm)	y (mm)	z (mm)	Z (max.)	cluster C	p (FWE corr.)	p (FDR corr.)	K (equiv.)	p (unc.)	peak T	p (FWE corr.)	p (FDR corr.)	Z (equiv.)	p (unc.)
p < 0.005	**LC (r)**	5	-37	-27	3.37	2	0.08	0.71	3	0.64	3.37	0.05	0.49	2.98	0.001
	LC (r)	3	-36	-19	3.37	2	0.09	0.71	2	0.71	3.37	0.05	0.49	2.98	0.001
	**SNr (r)**	13	-18	-9	4.77	5	0.02	0.07	89	0.01	4.77	0.05	0.37	3.88	< 0.001
	red Ncl (l)	-5	-17	-8	3.87	5	0.12	0.11	44	0.07	3.87	0.26	0.66	3.33	< 0.001
	red Ncl (r)	7	-22	-13	3.83	5	0.61	0.53	5	0.53	3.83	0.28	0.66	3.30	< 0.001
	red Ncl (r)	7	-18	-7	3.59	5	0.06	0.09	62	0.03	3.59	0.39	0.66	3.14	< 0.001
	SNr (l)	-12	-15	-13	3.47	5	0.29	0.24	21	0.19	3.47	0.46	0.66	3.05	0.001
p < 0.003	**LC (r)**	5	-37	-27	3.37	2	0.05	0.67	3	0.59	3.37	0.05	0.75	2.98	0.001
	LC (r)	3	-26	-19	3.37	2	0.06	0.67	2	0.67	3.37	0.05	0.75	2.98	0.001
	**SNr (r)**	13	-18	-9	4.77	9	0.02	0.12	70	0.01	4.77	0.05	0.49	4.77	< 0.001
	red Ncl (l)	-5	-17	-8	3.87	9	0.25	0.60	14	0.23	3.87	0.26	0.87	3.87	< 0.001
	red Ncl (r)	7	-22	-13	3.83	9	0.52	0.75	3	0.59	3.83	0.28	0.87	3.83	< 0.001
	red Ncl (r)	7	-18	-7	3.60	9	0.22	0.60	16	0.20	3.60	0.39	0.87	3.59	< 0.001
	SNr (l)	-12	-15	-13	3.47	9	0.32	0.60	10	0.31	3.47	0.46	0.87	3.47	0.001
	red Ncl (l)	-3	-21	-7	3.34	9	0.45	0.72	5	0.48	3.34	0.54	0.87	3.34	0.001
	red Ncl (r)	3	-18	-8	3.33	9	0.34	0.60	9	0.34	3.33	0.55	0.87	3.33	0.001

	region	x	y	z	Z	C	p	p	K	p	T	p	p	Z	p
	red Ncl (l)	-9	-18	-7	3.19	9	0.57	0.75	2	0.67	3.19	0.63	0.88	3.19	0.002
	VDC (r)	2	-22	-8	3.10	9	0.62	0.78	1	0.78	3.10	0.69	0.93	3.10	0.003

Supplementary Table 4. Activations in older subjects for task-related salience with applying a **'brainstem mask'** and small volume image of **'LC meta mask'** or **'SNredVTA mask'** cluster threshold p < 0.005 and p < 0.003 with no adjusted FDRc.

(2) task related salience: reversal > no reversal

older adults > younger adults

						cluster					peak				
	region	x (mm)	y (mm)	z (mm)	Z (max.)	C	p (FWE corr.)	p (FDR corr.)	K (equiv.)	p (unc.)	T	p (FWE corr.)	p (FDR corr.)	Z (equiv.)	p (unc.)
p < 0.005	**LC** (r)	4	-38	-28	3.02	1	0.07	0.63	4	0.63	3.02	0.05	0.77	2.88	0.002
	red Ncl (l)	-3	-21	-7	3.18	3	0.68	0.74	2	0.74	3.18	0.43	0.55	3.01	0.001
	VDC (l)	-9	-18	-7	3.18	3	0.48	0.74	10	0.42	3.18	0.43	0.55	3.01	0.001
	red Ncl (r)	3	-21	-7	2.83	3	0.68	0.74	2	0.74	2.83	0.69	0.76	2.70	0.003
p < 0.003	**LC (r)**	4	-38	-28	3.02	1	0.06	0.81	1	0.81	2.88	0.05	0.71	2.88	0.001
	red Ncl (l)	-3	-21	-7	3.18	2	0.57	0.81	1	0.81	3.18	0.43	0.60	3.01	0.001
	VDC (l)	-9	-18	-7	3.18	2	0.41	0.81	6	0.49	3.18	0.43	0.60	3.01	0.001

Supplemental Table 5. Activations in older > younger subjects for task-related salience with applying a **'brainstem mask'** and small volume image of **'LC meta mask'** or **'SNredVTA mask'** cluster threshold p < 0.005 and p < 0.003 with no adjusted FDRc.

younger adults **(2) task related salience: reversal > no reversal**

	region	x (mm)	y (mm)	z (mm)	Z (max.)	cluster C	p (FWE corr.)	p (FDR corr.)	K (equiv.)	p (unc.)	peak T	p (FWE corr.)	p (FDR corr.)	Z (equiv.)	p (unc.)
p < 0.005	SNc (l)	-10	-24	-13	4.34	6	0.25	0.56	27	0.19	4.34	0.07	0.54	3.75	0.001
	SNr (r)	4	-17	-17	3.46	6	0.65	0.83	3	0.68	3.46	0.36	0.78	3.12	0.001
	red Ncl (r)	8	-19	-10	3.44	6	0.08	0.34	60	0.06	3.44	0.36	0.78	3.12	0.001
	VDC (r)	4	-14	-7	3.02	6	0.72	0.83	1	0.83	3.02	0.64	0.80	2.78	0.003
	red Ncl (r)	5	-16	-12	2.85	6	0.72	0.83	1	0.83	2.85	0.74	0.88	2.64	0.004
	SNc (l)	-10	-19	-13	2.85	6	0.65	0.83	3	0.68	2.85	0.74	0.88	2.64	0.004
p < 0.003	SNc (l)	-10	-24	-13	4.34	8	0.21	0.80	18	0.22	4.34	0.07	0.65	3.75	< 0.001
	SNr (r)	4	-17	-17	3.46	8	0.50	0.80	3	0.63	3.46	0.36	0.94	3.12	< 0.001
	red Ncl. (r)	8	-19	-10	3.44	8	0.32	0.80	10	0.36	3.44	0.36	0.94	3.11	< 0.001
	SNr (r)	10	-22	-12	3.43	8	0.32	0.80	10	0.36	3.43	0.37	0.94	3.10	< 0.001
	SNr (r)	9	-18	-12	3.31	8	0.38	0.80	7	0.45	3.32	0.44	0.94	3.01	0.001
	red Ncl. (r)	5	-18	-10	3.14	8	0.53	0.80	2	0.71	3.14	0.55	0.94	2.87	0.002
	VDC (r)	4	-14	-7	3.02	8	0.58	0.80	1	0.80	3.02	0.64	0.96	2.78	0.003
	SNr (r)	11	-16	-14	3.00	8	0.58	0.80	1	0.80	3.00	0.64	0.96	2.77	0.003

Supplemental Table 6. Activations in younger subjects for task-related salience with applying a **‘brainstem mask’** and small volume image of **‘LC meta mask’** or **‘SNredVTA mask’** cluster threshold p < 0.005 and p < 0.003 with no adjusted FDRc.

(3) memory performance: remembered > not remembered

older adults

						cluster					peak				
	region	x (mm)	y (mm)	z (mm)	Z (max.)	C	p (FWE corr.)	p (FDR corr.)	K (equiv.)	p (unc.)	T	p (FWE corr.)	p (FDR corr.)	Z (equiv.)	p (unc.)
p < 0.005	**LC (r)**	5	-38	-25	3.64	1	0.07	0.53	5	0.53	3.64	0.03	0.21	3.17	< 0.001
p < 0.003	**LC (r)**	5	-38	-25	3.64	1	0.05	0.59	3	0.59	3.64	0.03	0.31	3.17	< 0.001

Supplemental Table 7. Activations in older subjects for memory performance with applying a **'brainstem mask'** and small volume image of **'LC meta mask'** cluster threshold p < 0.005 and p < 0.003 with no adjusted FDRc.

older > younger adults

						cluster					peak				
	region	x (mm)	y (mm)	z (mm)	Z (max.)	C	p (FWE corr.)	p (FDR corr.)	K (equiv.)	p (unc.)	T	p (FWE corr.)	p (FDR corr.)	Z (equiv.)	p (unc.)
p < 0.005	**LC (r)**	5	-38	-25	3.42	1	0.08	0.67	3	0.67	3.42	0.02	0.17	3.22	< 0.001
p < 0.003	**LC (r)**	5	-38	-25	3.42	1	0.05	0.70	2	0.70	3.42	0.02	0.26	3.22	< 0.001

Supplemental Table 8. Activations in older > younger subjects for memory performance with applying a **'brainstem mask'** and small volume image of **'LC meta mask'** cluster threshold p < 0.005 and p < 0.003 with no adjusted FDRc.

(3) memory performance: remembered > not remembered

younger adults

	region	x (mm)	y (mm)	z (mm)	Z (max.)	C	p (FWE corr.)	p (FDR corr.)	K (equiv.)	p (unc.)	T	p (FWE corr.)	p (FDR corr.)	Z (equiv.)	p (unc.)
						cluster					peak				
p < 0.005	SNr (l)	-11	-23	-17	3.23	2	0.74	0.82	1	0.82	3.23	0.51	0.66	2.95	0.002
	SNr (l)	-12	-24	-15	3.01	2	0.66	0.82	3	0.67	3.01	0.65	0.66	2.77	0.003
p < 0.003	SNr (l)	-11	-23	-17	3.23	2	0.59	0.80	1	0.80	3.23	0.51	0.95	2.95	0.002
	SNr (l)	-12	-24	-15	3.01	2	0.59	0.80	1	0.80	3.01	0.65	0.95	2.77	0.003

Supplemental Table 9. Activations in younger subjects for memory performance with applying a **'brainstem mask'** and small volume image of **'SNredVTA mask'** cluster threshold p < 0.005 and p < 0.003 with no adjusted FDRc.

younger > older adults

	region	x (mm)	y (mm)	z (mm)	Z (max.)	C	p (FWE corr.)	p (FDR corr.)	K (equiv.)	p (unc.)	T	p (FWE corr.)	p (FDR corr.)	Z (equiv.)	p (unc.)
						cluster					peak				
p < 0.005	SNc (r)	9	-26	-16	3.00	4	0.55	0.83	7	0.50	3.00	0.56	0.82	2.86	0.002
	red Ncl. (l)	-6	-24	-12	2.92	4	0.69	0.83	2	0.74	2.92	0.63	0.82	2.79	0.003
	VTA (l)	-3	-24	-19	2.90	4	0.73	0.83	1	0.83	2.90	0.64	0.82	2.77	0.003
	SNr (l)	-11	-23	-17	2.79	4	0.73	0.83	1	0.83	2.79	0.73	0.82	2.67	0.004
p < 0.003	SNc (r)	9	-26	-16	3.00	3	0.50	0.80	3	0.63	3.00	0.56	0.95	2.86	0.002
	red Ncl. (l)	-6	-24	-12	2.92	3	0.58	0.80	1	0.80	2.92	0.63	0.95	2.79	0.003
	VTA (l)	-3	-24	-19	2.90	3	0.58	0.80	1	0.80	2.90	0.64	0.95	2.77	0.003

Supplemental Table 10. Activations in younger > older subjects for memory performance with applying a **'brainstem mask'** and small volume image of **'LC meta mask'** or **'SNredVTA mask'** cluster threshold p < 0.005 and p < 0.003 with no adjusted FDRc.

(4) emotional memory performance: remembered before loss > not remembered before loss

older adults

	region	x (mm)	y (mm)	z (mm)	Z (max.)	C (cluster)	p (FWE corr.)	p (FDR corr.)	K (equiv.)	p (unc.)	T (peak)	p (FWE corr.)	p (FDR corr.)	Z (equiv.)	p (unc.)
p < 0.005	**LC (r)**	5	-38	-24	4.38	1	0.07	0.59	4	0.59	4.38	0.01	0.04	3.65	< 0.001
p < 0.003	**LC (r)**	5	-38	-24	4.38	1	0.06	0.67	2	0.67	4.38	0.01	0.07	3.65	< 0.001

Supplemental Table 11. Activations in older subjects for emotional memory performance with applying a **‘brainstem mask’** and small volume image of **‘LC meta mask’** cluster threshold p < 0.005 and p < 0.003 with no adjusted FDRc.

older > younger adults

	region	x (mm)	y (mm)	z (mm)	Z (max.)	C (cluster)	p (FWE corr.)	p (FDR corr.)	K (equiv.)	p (unc.)	T (peak)	p (FWE corr.)	p (FDR corr.)	Z (equiv.)	p (unc.)
p < 0.005	**LC (r)**	5	-38	-24	3.46	1	0.09	0.74	2	0.74	3.46	0.02	0.15	3.25	< 0.001
p < 0.003	**LC (r)**	5	-38	-24	3.46	1	0.05	0.70	2	0.70	3.46	0.02	0.23	3.25	< 0.001

Supplemental Table 12. Activations in older > younger subjects for emotional memory performance with applying a **‘brainstem mask’** and small volume image **‘LC meta mask’** cluster threshold p < 0.005 and p < 0.003 with no adjusted FDRc.

(4) emotional memory performance: remembered before loss > not remembered before loss

younger

	cluster										peak				
	region	x (mm)	y (mm)	z (mm)	Z (max.)	C	p (FWE corr.)	p (FDR corr.)	K (equiv.)	p (unc.)	T	p (FWE corr.)	p (FDR corr.)	Z (equiv.)	p (unc.)
p < 0.005	SNr (l)	-7	-24	-11	3.86	6	0.53	0.73	8	0.45	3.86	0.19	0.54	3.42	< 0.001
	SNc (l)	-11	-23	-17	3.44	6	0.70	0.73	2	0.73	3.44	0.39	0.54	3.12	< 0.001
	VTA (l)	-4	-22	-16	3.28	6	0.67	0.73	3	0.66	3.28	0.49	0.54	2.98	0.001
	red Ncl. (r)	5	-20	-11	3.26	6	0.55	0.73	7	0.48	3.26	0.50	0.54	2.97	0.001
	SNc (r)	10	-25	-15	3.22	6	0.63	0.73	4	0.60	3.22	0.53	0.54	2.93	0.003
	red Ncl. (l)	-2	-21	-12	3.12	6	0.60	0.73	5	0.55	3.12	0.60	0.54	2.86	0.002
p < 0.003	SNr (l)	-7	-24	-11	3.86	6	0.44	0.79	5	0.50	3.86	0.19	0.78	3.42	< 0.001
	SNc (l)	-11	-23	-17	3.44	6	0.55	0.79	2	0.69	3.44	0.39	0.78	3.12	0.001
	VTA (l)	-4	-22	-16	3.28	6	0.55	0.79	2	0.69	3.28	0.49	0.78	2.98	0.001
	red Ncl. (r)	5	-20	-11	3.26	6	0.47	0.79	4	0.55	3.26	0.50	0.78	2.97	0.001
	SNc (r)	10	-25	-15	3.22	6	0.47	0.79	4	0.55	3.22	0.53	0.78	2.93	0.002
	red Ncl. (l)	-2	-21	-12	3.12	6	0.60	0.79	1	0.79	3.12	0.60	0.78	2.89	0.002

Supplemental Table 13. Activations in younger subjects for emotional memory performance with applying a **‘brainstem mask’** and small volume image of **‘LC meta mask’** or **‘SNredVTA mask’** cluster threshold p < 0.005 and p < 0.003 with no adjusted FDRc.

(4) emotional memory performance: remembered before loss > not remembered before loss

younger > older adults

						cluster					peak				
	region	x (mm)	y (mm)	z (mm)	Z (max.)	C	p (FWE corr.)	p (FDR corr.)	K (equiv.)	p (unc.)	T	p (FWE corr.)	p (FDR corr.)	Z (equiv.)	p (unc.)
p < 0.005	red Ncl. (l)	-4	-24	-12	2.96	3	0.69	0.82	2	0.74	2.96	0.60	0.91	2.82	0.002
	VDC (l)	-11	-24	-17	2.81	3	0.73	0.82	1	0.82	2.81	0.72	0.91	2.69	0.004
	SNr (l)	-10	-23	-18	2.73	3	0.73	0.82	1	0.82	2.73	0.77	0.91	2.62	0.004
p < 0.003	red Ncl (l)	-4	-24	-12	2.96	1	0.59	0.80	1	0.80	2.96	0.60	0.84	2.82	0.002

Supplemental Table 14. Activations in younger > older subjects for emotional memory performance with applying a **‘brainstem mask’** and small volume image **‘LC meta mask’** or **‘SNredVTA mask’** cluster threshold p < 0.005 and p < 0.003 with no adjusted FDRc.

cortical activations

(1) emotional salience: loss feedback > gain feedback

older adults

						cluster					peak				
	region	x (mm)	y (mm)	z (mm)	Z (max.)	C	p (FWE corr.)	p (FDR corr.)	K (equiv.)	p (unc.)	T	p (FWE corr.)	p (FDR corr.)	Z (equiv.)	p (unc.)
p < 0.005	**MTG (r)**	57	-35	-3	7.74	10	0	< 0.001	4935	< 0.001	7.74	0.03	0.19	5.27	< 0.001
	**LiG (r)**	17	-49	-2	6.62	10	< 0.001	< 0.001	481	< 0.001	6.62	0.28	0.23	4.81	< 0.001
	**FuG (r)**	39	-39	-23	6.37	10	< 0.001	< 0.001	2869	< 0.001	6.37	0.40	0.23	4.70	< 0.001
	WM	28	-62	-12	5.38	10	0.05	0.02	259	< 0.001	5.38	0.95	0.47	4.22	< 0.001
	CBM	-29	-62	-25	5.24	10	0.016	0.03	199	0.001	5.24	0.98	0.51	4.15	< 0.001
	ITG (l)	-43	-61	-5	5.08	10	0.19	0.04	199	0.001	5.08	0.99	0.59	4.06	< 0.001
	**MTG (l)**	-50	-34	-1	4.79	10	< 0.001	< 0.001	1451	< 0.001	4.79	0.99	0.64	3.90	< 0.001
	**CAL (l)**	-15	-70	5	4.60	10	0.01	0.004	325	< 0.001	4.60	0.99	0.65	3.78	< 0.001
	WM	11	-82	10	4.36	10	< 0.001	< 0.001	527	< 0.001	4.36	1	0.68	3.64	< 0.001
	CAL (l)	-7	-87	5	4.12	10	0.17	0.04	196	0.001	4.12	1	0.70	3.49	< 0.001

Supplemental Table 15. Activations in older subjects for emotional salience with applying a 'grey matter mask', cluster threshold p < 0.005 with adjusted FDRc = 173.

(1) emotional salience: loss feedback > gain feedback

older adults > younger adults

						cluster					peak				
	region	x (mm)	y (mm)	z (mm)	Z (max.)	C	p (FWE corr.)	p (FDR corr.)	K (equiv.)	p (unc.)	T	p (FWE corr.)	p (FDR corr.)	Z (equiv.)	p (unc.)
$p < 0.005$	**CBM**	20	-46	-25	4.99	3	0.07	0.04	272	< 0.001	4.99	0.51	0.92	4.46	< 0.001
	**MTG (l)**	-61	-42	-1	4.54	3	0.001	0.004	531	< 0.001	4.54	0.91	0.92	4.12	< 0.001
	**MTG (r)**	62	-12	-14	4.29	3	0.004	0.04	433	< 0.001	4.29	0.99	0.92	3.93	< 0.001
$p < 0.05$	**HPC (l)**	-30	-22	-17	3.94	31	0.008	0.007	1568	< 0.001	3.94	0.47	0.56	3.65	< 0.001

Supplemental Table 16. Activations in older > younger subjects for emotional salience with applying a 'grey matter mask', cluster threshold $p < 0.005$ with adjusted FDRc = 253; with applying a 'hippocampus amygdala mask', cluster threshold $p < 0.05$ no adjusted FDRc.

younger adults

						cluster					peak				
	region	x (mm)	y (mm)	z (mm)	Z (max.)	C	p (FWE corr.)	p (FDR corr.)	K (equiv.)	p (unc.)	T	p (FWE corr.)	p (FDR corr.)	Z (equiv.)	p (unc.)
$p < 0.005$	**CAL (l)**	-7	-71	8	6.93	3	< 0.001	< 0.001	1752	< 0.001	6.93	0.04	0.05	5.21	< 0.001
	**MTG (r)**	50	-31	-3	5.32	3	< 0.001	< 0.001	1271	< 0.001	5.32	0.76	0.27	4.36	< 0.001
	**CAL (r)**	7	-65	8	5.23	3	< 0.001	< 0.001	1311	< 0.001	5.23	0.82	0.27	4.31	< 0.001

Supplemental Table 17. Activations in younger subjects for emotional salience with applying a 'grey matter mask', cluster threshold $p < 0.005$ with adjusted FDRc = 422.

(2) task related salience: reversal > no reversal

older adults

						cluster					peak				
	region	x (mm)	y (mm)	z (mm)	Z (max.)	C	p (FWE corr.)	p (FDR corr.)	K (equiv.)	p (unc.)	T	p (FWE corr.)	p (FDR corr.)	Z (equiv.)	p (unc.)
p < 0.005	**MTG (r)**	68	-39	0	8.14	5	0	< 0.001	3894	< 0.001	8.14	0.01	0.07	5.41	< 0.001
	**PCUN (r)**	21	-54	5	6.59	5	0.0002	< 0.001	512	0.31	6.59	0.31	0.32	4.80	< 0.001
	unknown	-61	-63	-10	5.85	5	0.07	0.04	222	0.75	5.85	0.75	0.70	4.56	< 0.001
	**MTG (l)**	-68	-35	-1	5.70	5	0.003	0.001	373	0.83	5.70	0.83	0.70	4.38	< 0.001
	**CAL (l)**	-9	-80	13	4.74	5	< 0.001	< 0.001	559	0.99	4.74	1	0.70	3.87	< 0.001

Supplemental Table 18. Activations in older subjects for task related salience with applying a ‘grey matter mask’, cluster threshold p < 0.005 with adjusted FDRc = 181.

older adults > younger adults

						cluster					peak				
	region	x (mm)	y (mm)	z (mm)	Z (max.)	C	p (FWE corr.)	p (FDR corr.)	K (equiv.)	p (unc.)	T	p (FWE corr.)	p (FDR corr.)	Z (equiv.)	p (unc.)
p < 0.005	**PCUN (l)**	-7	-62	14	4.44	1	< 0.001	< 0.001	739	< 0.001	4.96	0.55	0.93	4.44	< 0.001

Supplemental Table 19. Activations in older > younger subjects for task related salience with applying a ‘grey matter mask’, cluster threshold p < 0.005 with adjusted FDRc = 724.

(2) task related salience: reversal > no reversal

younger adults

						cluster					peak				
	region	x (mm)	y (mm)	z (mm)	Z (max.)	C	p (FWE corr.)	p (FDR corr.)	K (equiv.)	p (unc.)	T	p (FWE corr.)	p (FDR corr.)	Z (equiv.)	p (unc.)
p < 0.005	**STG (r)**	49	-22	-8	6.61	4	< 0.001	< 0.001	4252	< 0.001	6.61	0.09	0.13	5.05	< 0.001
	**STG (l)**	-53	-33	1	5.54	4	< 0.001	< 0.001	2073	< 0.001	5.54	0.60	0.20	4.50	< 0.001
	**MTG (l)**	-59	-30	-15	5.28	4	0.04	0.02	301	< 0.001	5.28	0.80	0.22	4.34	< 0.001
	**EC (l)**	-27	-3	-40	4.58	4	0.15	0.05	225	< 0.001	4.58	1	0.35	3.91	< 0.001

Supplemental Table 20. Activations in younger subjects for task related salience with applying a 'grey matter mask', cluster threshold p < 0.005 with adjusted FDRc = 195.

(4) emotional memory performance: remembered before loss > not remembered before loss

older adults > younger adults

						cluster					peak				
	region	x (mm)	y (mm)	z (mm)	Z (max.)	C	p (FWE corr.)	p (FDR corr.)	K (equiv.)	p (unc.)	T	p (FWE corr.)	p (FDR corr.)	Z (equiv.)	p (unc.)
p < 0.005	**LiG (l)**	-20	-71	-14	3.93	1	0.009	0.01	358	< 0.001	3.93	0.99	0.92	3.64	< 0.001

Supplemental Table 21. Activations in older > younger subjects for emotional memory performance with applying a 'grey matter mask', cluster threshold p < 0.005 with adjusted FDRc = 289.

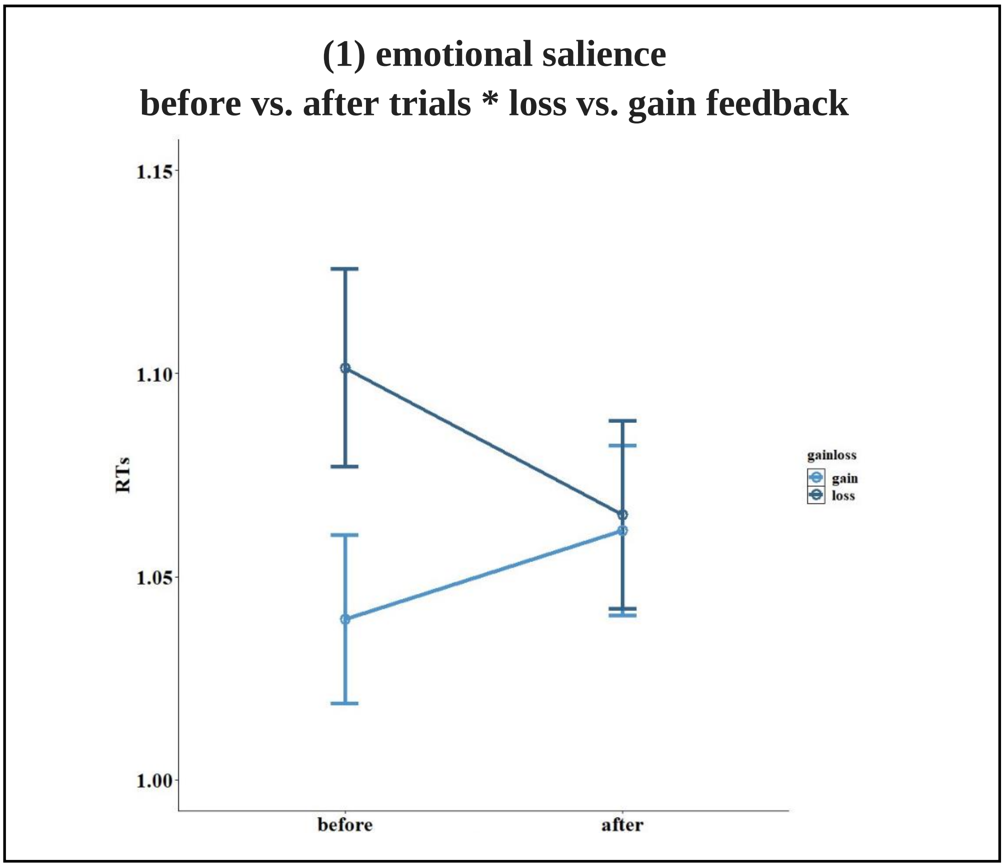

Supplementary Figure 5. Mean RTs for loss (dark blue) and gain (light blue) feedback after (x-axis, left) and before (x-axis, right) trials across age-groups for (1) emotional salience. RTs slowed down after gain feedback but sped up after loss feedback (loss feedback: before trials (M = 1.10, SD = 0.03), after trials (M = 1.07, SD = 0.02), gain feedback: before trials (M = 1.04, SD = 0.02), after trials (M = 1.06, SD = 0.02); $F(1,48) = 19.40$, $p < 0.001$, partial $\eta^2 = .29$).

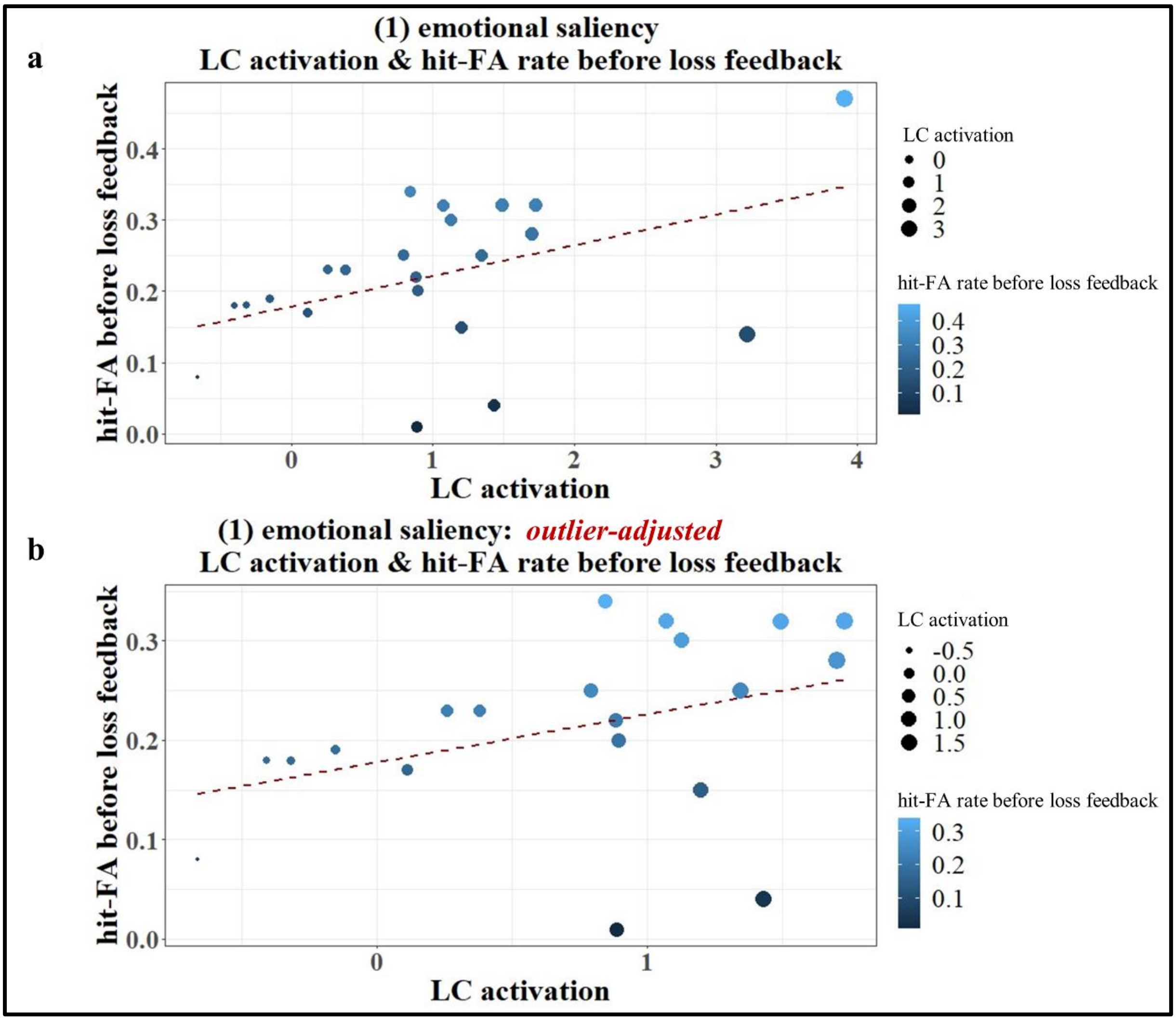

Supplementary Figure 6. Relation between LC activation (x-axis) and memory performance for stimuli before loss feedback (y-axis) for older adults is shown for a) all older adults (N = 22; r (20) = .37, p = 0.04) and corrected for b) outliers (N = 20; r (18) = .42, p = 0.11). After the correction for outliers, there is no longer a significant relationship between higher LC activation and better memory performance for stimuli before loss feedback for older adults. The strength of LC activation per older individual is indicated with black circles (-0.5-3), and the hit-FA rate is indicated in blue gradations (0.1-0.4) along a fitted regression line (red dashed line).

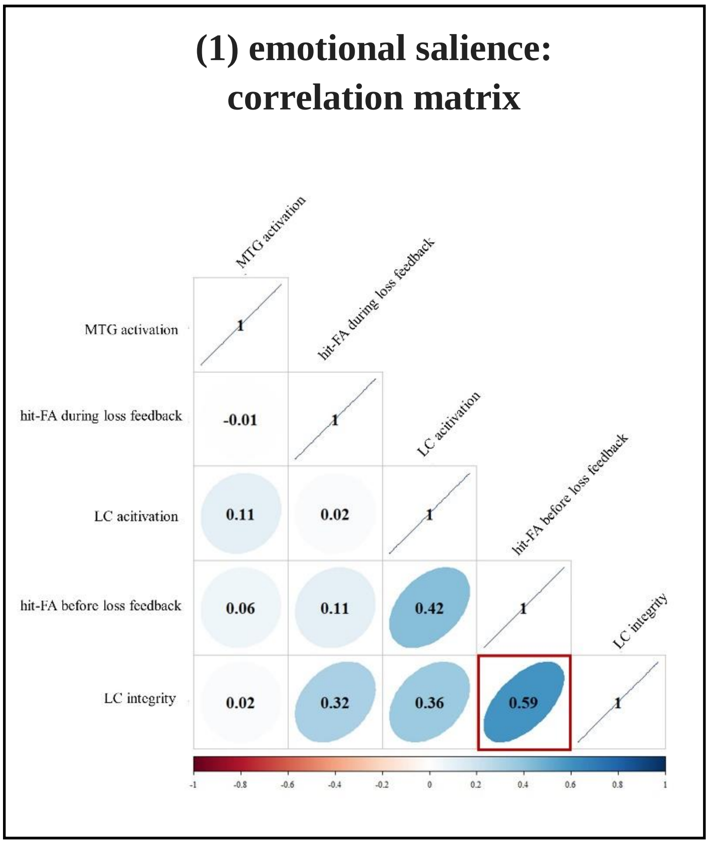

Supplementary Figure 7. Spearman´s rank correlation matrix for **emotional salience** in older adults displays the strength of the relationship (red to blue) of correlation coefficients for significant averaged LC activation with voxel-cut off of 0.005, averaged MTG activation, LC integrity and memory performance as hit-FA rate before loss feedback and the difference in hit-FA rate from loss feedback before vs. after trials. The *ellipsis* indicates the direction of the correlation, the *number* the strength of the correlation, and the *red rectangle* significant correlations. Correlations were outlier corrected (based on LC activation) and adjusted with Bonferroni corrections for multiple comparisons. Increased LC integrity led to better memory performance (r (18) = 0.59, p = 0.004). Removed outliers: ID 1 & 3.

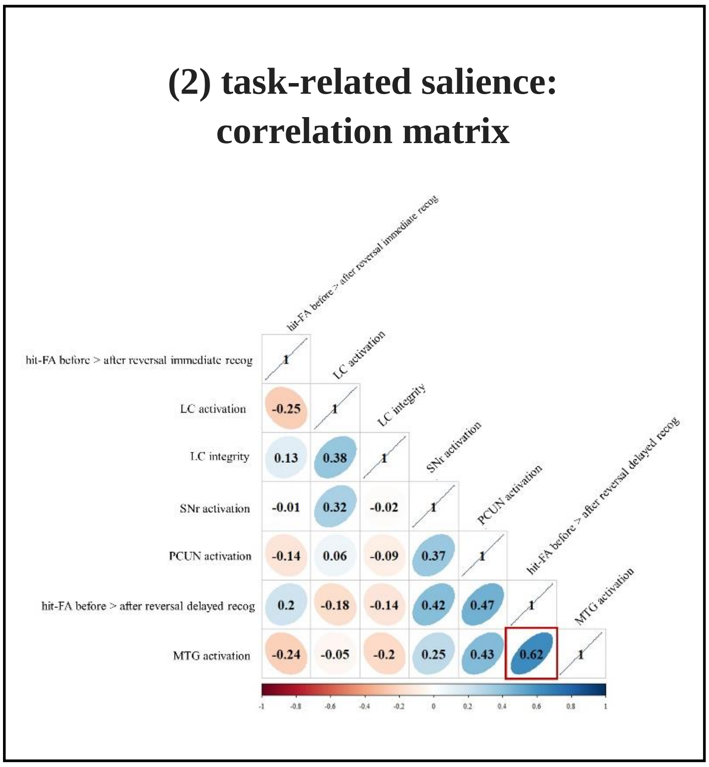

Supplementary Figure 8. Spearman´s rank correlation for **task-related salience** in older adults displays the strength of the relationship (red to blue) of correlation coefficients for significant averaged LC activation and SNr activation with voxel-cut off of 0.005, averaged MTG and PCUN activation, LC integrity and memory performance as the difference in hit-FA rate from reversals before vs. after three trials for immediate and delayed recognition. The *ellipsis* indicates the direction of the correlation, the *number* the strength of the correlation, and the *red rectangle* significant correlations. Correlations were outlier corrected (based on LC activation) and adjusted with Bonferroni corrections for multiple comparisons. Increased MTG activation led to better memory performance (r (16) = 0.62, p = 0.009). Removed outliers: ID 1, 3, 19, 21.

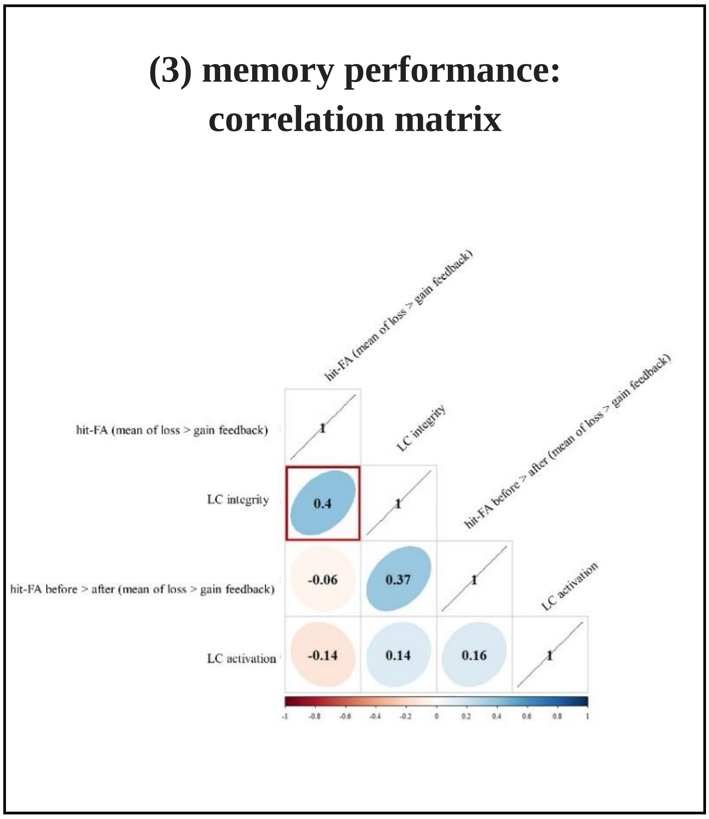

Supplementary Figure 9. Spearman´s rank correlation for **memory performance** in older adults displays the strength of the relationship (red to blue) of correlation coefficients for significant LC activation with voxel-cut off of 0.005, LC integrity and memory performance as hit-FA rate of mean of loss vs. gain feedback and the difference in hit-FA rate from loss vs gain feedback before vs. after trials. The *ellipsis* indicates the direction of the correlation, the *number* the strength of the correlation, and the *red rectangle* significant correlations. Correlations were outlier corrected (based on LC activation) and adjusted with Bonferroni corrections for multiple comparisons. Higher LC integrity was associated with better memory performance (r (19) = 0.40, p = 0.03). Removed outliers: ID 3.

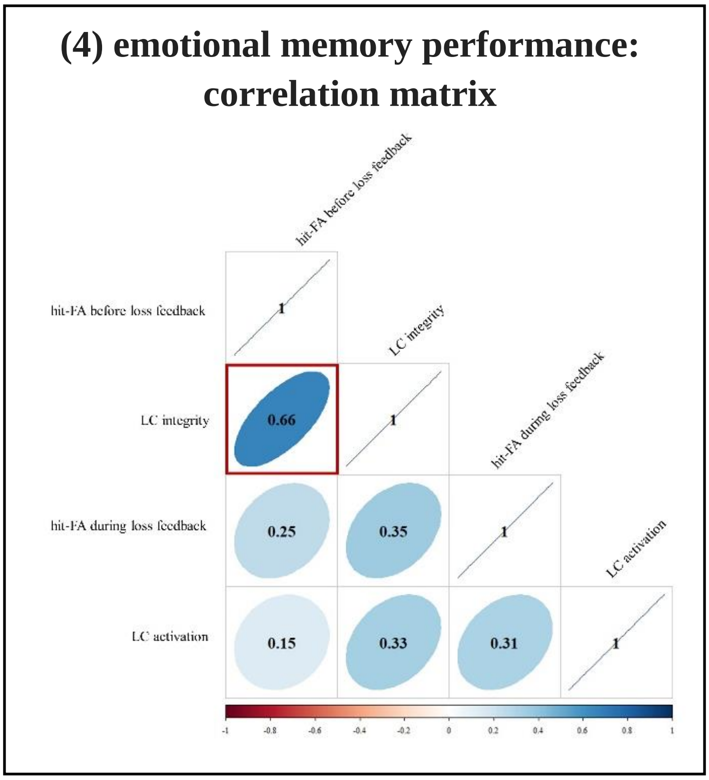

Supplementary Figure 10. Spearman´s rank correlation for **emotional memory performance** in older adults displays the strength of the relationship (red to blue) of correlation coefficients for significant LC activation with voxel-cut off of 0.005, LC integrity and memory performance as hit-FA rate after loss feedback and the difference in hit-FA rate from loss feedback before vs. after trials. The *ellipsis* indicates the direction of the correlation, the *number* the strength of the correlation, and the *red rectangle* significant correlations. Correlations were outlier corrected (based on LC activation) and adjusted with Bonferroni corrections for multiple comparisons. However, increased LC integrity and better memory performance based on the outlier correction of LC activation during emotional memory performance should not be interpreted in contrast to emotional salience, as outlier were corrected based on LC activation and not LC integrity. Removed outliers: ID 9 & 14.

Time course of the effect size

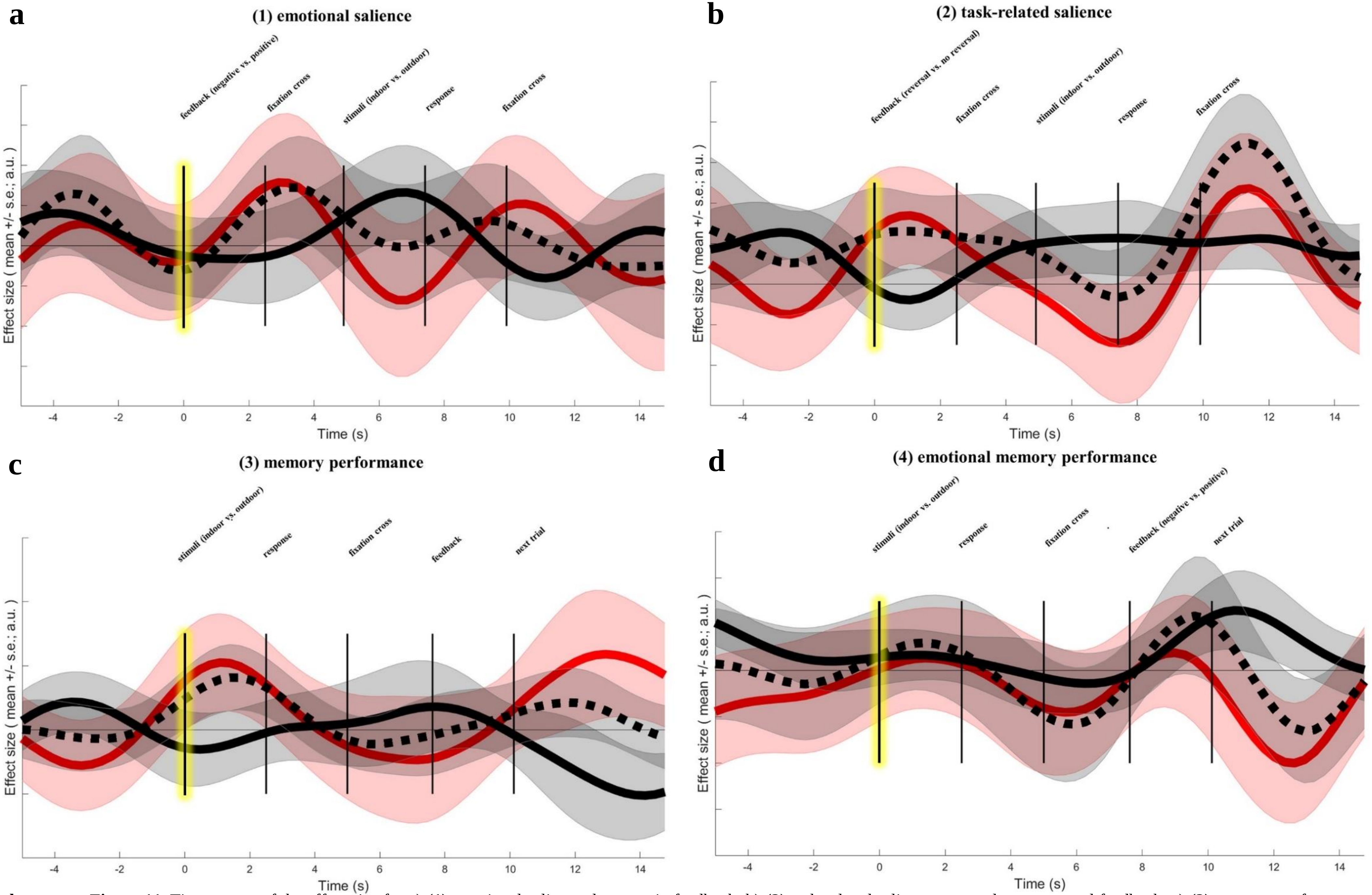

Supplementary Figure 11. Time course of the effect size for a) **(1)** emotional salience: loss > gain feedback, b) **(2)** task-related salience: reversal > no reversal feedback, c) **(3)** memory performance: remembered > not remembered and d) **(4)** emotional memory performance: remembered before loss feedback > not remembered before loss feedback for Locus Coeruleus (LC) activations (voxel cut-offs of $p < 0.005$). Beta values per contrast (a-d) for older adults (dotted black line) and younger adults (solid black line) and (a-d) the coefficient estimates for older adults > younger adults (solid red line) are plotted; shaded areas +/- 1 SE. Yellow lines indicate the onset of relevant task events.

Reversal reinforcement learning task

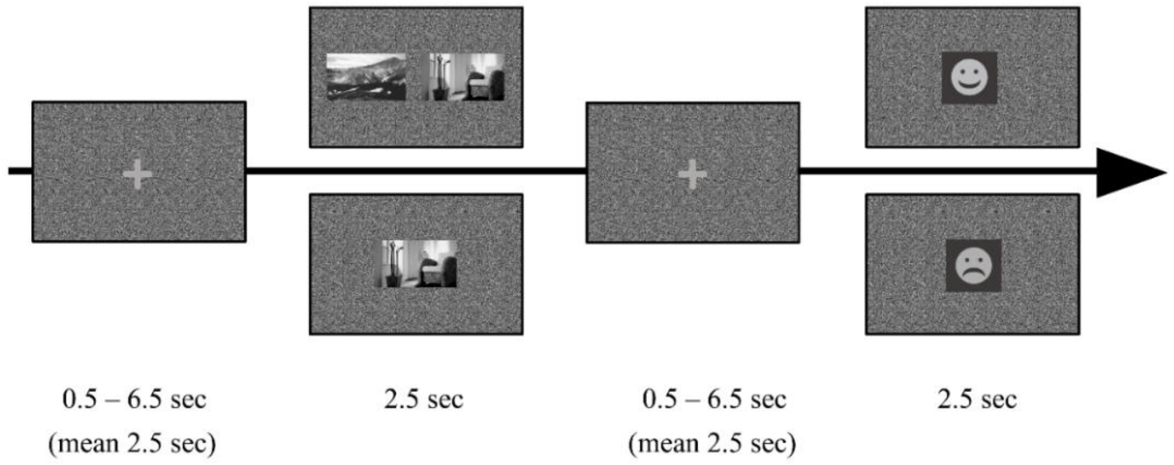

Supplementary Figure 12. Reversal reinforcement learning task (adapted from Hämmerer et al., 2018).

Sample description

	older adults	younger adults
**number of subjects**	22	28
**age** (mean±SD)	67.68 ± 5.68	23.14 ± 3.18
**gender (F/M)**	12 / 10	16 / 12
**Raven´s matrices** (mean±SD)	14.09 ± 2.43	16.26 ± 1.37

Supplementary Table 22. Overview of sample description. A shortened version of Raven's matrices was used as a measure of fluid intelligence (older adults (N = 22); younger adults (N = 19). The values indicate the correct answers from a total of 18 matrices.

Supplementary Results 1

Results

A reversal learning task with trial-unique scene stimuli was used to assess behavioural and brain responses to salience. This task included reversals as task-related salient events and negative versus positive feedback as emotionally salient events. By controlling response options on half of the trials (see *Methods* for details), task-related salience and emotional salience were decorrelated and could be separately evaluated.

Behavioural results

Regarding memory effects of **(1) emotional salience,** there was no or interaction between *loss vs. gain feedback* and *age group* ($F(1,48) = 0.013$, $p = 0.911$ [younger: loss feedback (M = 0.20, SD = 0.02), gain feedback (M = 0.18, SD = 0.02); older: loss feedback (M = 0.21, SD = 0.02), gain feedback (M = 0.20, SD = 0.02)]), as well as no interaction between *trials before vs trials after feedback* and *age group* ($F(1,48) = 0.18$, $p = 0.693$ [younger: trials before (M = 0.19, SD = 0.02), trials after (M = 0.19, SD = 0.02); older: trials before (M = 0.20, SD = 0.02), trials after (M = 0.20, SD = 0.02)]).

Reaction times (RTs) for (1) emotional salience

Regarding RTs related to **(1) emotional salience**, RTs averaged across before and after trials were significantly slower related to gain feedback (M = 1.05, SD = 0.02) as compared to loss feedback (M = 1.09, SD = 0.02), $F(1,48) = 31.29$, $p < 0.0001$, partial η^2 = .40. There were significant interactions between *loss vs gain feedback* and *age group* ($F(1,48) = 9.40$, $p = 0.04$, partial η^2 = .164, [younger: loss feedback (M = 0.99, SD = 0.03), gain feedback (M = 0.98, SD = 0.03), older: loss feedback (M = 1.18, SD = 0.04), gain feedback (M = 1.12, SD = 0.03) as well as between *number of stimuli* and *age group* ($F(1,48) = 5.34$, $p = 0.03$, partial η^2 = .10, [younger: single stimulus (M = 0.86, SD = 0.03), double stimuli (M = 1.12, SD = 0.04), older: single stimulus (M = 1.07, SD = 0.03), double stimuli (M = 1.23, SD = 0.04)]). RTs were slower during loss as compared to gain feedback. Older adults (M = 1.2, SD = 0.4) showed a longer RTs as compared to younger adults (M = .98, SD = 0.31), $F(1,48) = 12.94$, $p < 0.001$, partial η^2 = .212 during emotional salience. Likewise, RTs were slower on trials before the feedback (M = 1.07, SD = 0.02) was presented as compared to after the feedback (M = 1.06, SD = 0.02), $F(1,48) = 5.441$, $p = 0.024$, partial η^2 = .10, indicating that subjects have become faster over the task. RTs were also slower during the presentation of two stimuli (M = 1.17, SD = 0.03) as compared to one stimuli (M = 0.97, SD = 0.02)., $F(1,48) = 140.30$, $p < 0.001$, partial η^2 = .75. Additionally, there was a significant interaction between the *single vs. double stimuli*

and *loss vs. gain feedback*, $F(1,48) = 4.83$, $p = 0.03$, partial $\eta^2 = .09$: RTs were faster during single stimulus presentation and gain feedback [loss feedback: single stimulus (M = 0.98, SD = 0.02), double stimuli (M = 1.19, SD = 0.03), gain feedback: single stimulus (M = 0.95, SD = 0.02), double stimuli (M = 1.15, SD = 0.03). There was a significant three-way interaction between the *single vs double stimuli*, *before vs loss vs. gain feedback* and *age group,* $F(1,48) = 5.87$, $p = 0.02$, partial $\eta^2 = .12$: Younger adults were generally faster, RTs were also faster during gain feedback and single stimulus presentation (younger: single stimulus loss feedback (M = 0.86, SD = 0.03), single stimulus gain feedback (M = 0.86, SD = 0.03), double stimulus loss feedback (M = 1.12, SD = 0.04), double stimulus gain feedback (M = 1.09, SD = 0.03); older: single stimulus loss feedback (M = 1.09, SD = 0.04), single stimulus gain feedback (M = 1.04, SD = 0.03), double stimulus loss feedback (M = 1.26, SD = 0.04), double stimulus gain feedback (M = 1.21, SD = 0.04). Another significant three-way interaction between the *single vs double stimuli*, *before vs loss vs. gain feedback* and *before vs. after trials,* $F(1,48) = 12.28$, $p = 0.001$, partial $\eta^2 = .204$, indicated that, RTs were again faster during single stimulus presentation and gain feedback. However, for gain feedback, RTs were the same when presenting double stimuli both before and after trials (before: single stimulus loss feedback (M = 1, SD = 0.03), single stimulus gain feedback (M = 0.93, SD = 0.02), double stimulus loss feedback (M = 1.20, SD = 0.03), double stimulus gain feedback (M = 1.15, SD = 0.03); after: single stimulus loss feedback (M = 0.95, SD = 0.03), single stimulus gain feedback (M = 0.98, SD = 0.02), double stimulus loss feedback (M = 1.18, SD = 0.03), double stimulus gain feedback (M = 1.15, SD = 0.03). There was no significant interaction between *trials before vs. after* and the *age group* ($F(1,48) = .50$, $p = 0.49$, partial $\eta^2 = .01$, [younger: trials before (M = 0.97, SD = 0.03), trials after (M = 0.98, SD = 0.03), older: trials before (M = 1.16, SD = 0.04), trials after (M = 1.15, SD = 0.03)]). Regarding RTs related to **(2) task-related saliency,** older adults (M = 1.1, SD = 0.4) showed a longer RTs as compared to younger adults (M = .94, SD = 0.3), $F(1,48) = 18.20$, $p < 0.001$, partial $\eta^2 = .28$.

Supplementary Results 2

fMRI results

The analysis procedure described in 'fMRI results' resulted in no suprathreshold for a) **LC activation** in younger adults and younger > older adults for (**(1)** emotional salience, **(2)** task-related salience, **(3)** memory performance, **(4)** emotional memory performance)), b) for SNc, SNr, VTA and red nucleus activation in younger > older adults **(1)** emotional salience, **(2)** task-related salience**)** and older adults and older > younger adults **((3)** memory performance, **(4)**

emotional memory performance**)**. Also, no suprathreshold clusters for **cortical areas** were found in younger and older adults (**(3)** memory performance, **(4)** emotional memory performance), younger > older adults (**(1)** emotional salience, **(2)** task-related salience, **(3)** memory performance) and older > younger adults (**(3)** memory performance). Finally, no suprathreshold clusters **for subcortical** areas were found in younger and older adults (**(1)** emotional salience, **(2)** task-related salience, **(3)** memory performance, **(4)** emotional memory performance), younger > older adults **(1)** emotional salience, **(2)** task-related salience, **(3)** memory performance, **(4)** emotional memory performance), as well as older > younger adults (**(2)** task-related salience, **(3)** memory performance, **(4)** emotional memory performance)).

Activation in cortical areas

During **(1) loss > gain feedback** (see Supplementary Fig. 2; Table 15-17) higher left **CAL** [Younger adults: T = 6.93, pFDR < 0.01; Older adults: T = 4.60, pFDR < 0.01), and right **CAL** (Younger adults: T = 5.23, pFDR < 0.01) as well as right **FuG** (Older adults: T = 6.37, pFDR < 0.01) and right **LiG** (Older adults: T = 6.62, pFDR < 0.01) were observed. During **(2) reversal > no reversal feedback** (see Supplementary Fig. 3; Table 18-20) older adults showed stronger engagement of left **CAL** (T = 4.74, pFDR < 0.01). Similarly, as compared to younger adults, older adults also showed higher activation of the left **LiG** during **(4) later remembered stimuli followed by loss** as compared to not remembered stimuli followed by loss feedback (see Supplementary Fig. 4; Table 21) (Older > Younger adults: T = 3.93, pFDR < 0.01).

Additionally, for **(2)** task-related saliency in older adults, activation in MTG was significantly **correlated** with better memory performance for stimuli on trials immediately before a reversal in the delayed recognition (difference before and after trials), r (16) = .62, p < 0.009, indicating that stronger MTG activation is related to better memory performance before events of task-related salience (see Supplementary Fig. 8).

Sex differences

Regarding potential sex differences in **LC activation** we additionally investigated the main contrasts of interests: **1)** loss feedback > gain feedback as an indicator of **emotional salience** and **(2)** reversal feedback > no-reversal feedback as an indicator of **task-related salience**, **(3)** remembered stimuli > not remembered stimuli as in indicator of **memory performance** and **(4)** remembered stimuli before loss feedback > not remembered stimuli before loss feedback as an indicator of **emotional memory performance**. Activations in the brainstem were investigated using an inclusive brainstem mask (see section *Anatomical masks for second-level*

analyses). Given the small size of our target structures in the brainstem activations were assessed using anatomical masks of the LC. This described analysis procedure did not result in any suprathreshold clusters for brainstem in the contrasts listed above when examining sex differences, so unfortunately, we cannot report any results on sex differences.

Regarding potential sex differences in **behavioural memory performance**, we additionally run a repeated measures ANOVA for stimuli that occurred on trials before and after loss vs. gain feedback. There was no significant main effect of sex, $F(1,48) = 0.05$, $p = 0.83$. Likewise, when controlling for age and sex, there was no significant effect of sex, $F(1,46) = 0.05$, $p = 0.83$ and also no significant interaction between sex and age, $F(1,46) = 0.37$, $p = 0.55$.

Types of event-related GLMs	contrast of interest	fMRI results
**(1) emotional salience**	**loss feedback > gain feedback**	**younger adults**
		▪ right **MTG**
		**older adults**
		▪ left **LC** (Fig. 2a)
		▪ bilateral **MTG**
		**older > younger**
		▪ bilateral **LC** (Fig. 4a)
		▪ bilateral **MTG** (Fig.5a-b)
		▪ left **HPC** (Fig. 5c)
**(2) task-related salience**	**reversal feedback > no reversal feedback**	**younger adults**
		▪ left **MTG**
		▪ bilateral **STG**
		▪ left **EC**
		**older adults**
		▪ right **LC** (Fig. 2b)
		▪ right **SNr** (Fig. 3)
		▪ bilateral **MTG**
		▪ right **PCUN**
		**older > younger**
		▪ right **LC** (Fig. 4b)
		▪ left **PCUN**
**(3) memory performance**	**remembered > not remembered**	**younger adults**
		/
		**older adults**
		▪ right **LC** (Fig. 2c)
		**older > younger**
		▪ right **LC** (Fig. 4c)
**(4) emotional memory performance**	**remembered before loss feedback > not remembered before loss feedback**	**younger adults**
		/
		**older adults**
		▪ right **LC** (Fig. 2d)
		**older > younger**
		▪ right **LC** (Fig. 4d)

Supplementary Table 23. Four types of event-related GLMs with corresponding contrasts of interest and overview of the main fMRI results of younger, older and the group comparison older > younger adults. Additional fMRI results (e.g., CAL, FuG, LiG) are mentioned in Supplementary Results 2.

Supplementary Results 3

Age-related differences in 8 brainstem landmarks' mean functional images in MNI space. There were no age-related difference for the left 4th ventricle border (younger adults = 0.77, older adults = 0.73, t(1,48) = 0.52, p = 0.61), right 4th ventricle border (younger adults = 0.71, older adults = 0.68, t(1,48) = 0.37, p = 0.71), periaqueductal grey (younger adults = 0.68, older adults = 0.57, t(1,48) = 1.79, p = 0.08), perifastigial sulcus (younger adults = 0.54, older adults = 0.52, t(1,48) = 0.38, p = 0.71), left outline brainstem (younger adults = 0.38, older adults = 0.25, t(1,48) = 1.40, p = 0.17) as well as right outline brainstem (younger adults = 0.32, older adults = 0.27, t(1,48) = 0.53, p = 0.60). However, there were age-related difference for left nucleus ruber (younger adults = 0.79, older adults = 0.57, t(1,48) = 2.1, p = 0.04) and right nucleus ruber (younger adults = 0.82, older adults = 0.55, t(1,48) = 2.73, p = 0.01).
